# Graphene quantum dots rescue angiogenic retinopathy via blocking STAT3/Periostin/ERK signaling

**DOI:** 10.1186/s12951-022-01362-4

**Published:** 2022-04-02

**Authors:** Na Zhao, Xiao Gui, Qian Fang, Rui Zhang, Weiye Zhu, Haorui Zhang, Qing Li, Yukun Zhou, Jiawei Zhao, Xiao Cui, Guangping Gao, Huipeng Tang, Ni Shen, Taoyong Chen, Hongyuan Song, Wei Shen

**Affiliations:** 1 Department of Ophthalmology, Shanghai Changhai Hospital, Naval Medical University, Shanghai, 200433 China; 2 National Key Laboratory of Medical Immunology, Institute of Immunology, Naval Medical University, Shanghai, 200433 China; 3 Department of Ophthalmology, Shanghai General Hospital, Shanghai Jiao Tong University School of Medicine, Shanghai, 200080 China

**Keywords:** Retinal neovascularization, Oxygen induced retinopathy, Graphene quantum dots, Periostin, Cell cycle, RNA sequencing

## Abstract

**Background:**

Pathological retinal angiogenesis resulting from a variety of ocular diseases including oxygen induced retinopathy, diabetic retinopathy and ocular vein occlusion, is one of the major reasons for vision loss, yet the therapeutic option is limited. Multiple nanoparticles have been reported to alleviate angiogenic retinopathy. However, the adverse effect cannot be ignored due to the relatively large scale. Graphene quantum dots (GQDs) have shown potential in drug delivery and have been proved biocompatible. In this study, Graphene quantum dots are extensively investigated for their application in angiogenic retinopathy therapy.

**Results:**

We showed that GQDs were biocompatible nanomaterials in vitro and in vivo. The nanoparticles have a dose-dependent inhibitory effect on proliferation, migration, tube formation and sprouting of human umbilical vein endothelial cells (HUVECs). Further data show that GQDs could inhibit pathological retinal neovascularization in an oxygen-induced retinopathy (OIR) model. The data of RNA sequencing suggested that periostin is involved in this process. GQDs inhibit the expression of periostin via STAT3, and further regulated cell cycle-related protein levels through ERK pathway. The signaling pathway was conformed in vivo using OIR mouse model.

**Conclusions:**

The present study indicated that GQDs could be a biocompatible anti-angiogenic nanomedicine in the treatment of pathological retinal neovascularization via disrupting periostin/ERK pathway and subsequent cell cycle.

**Graphical Abstract:**

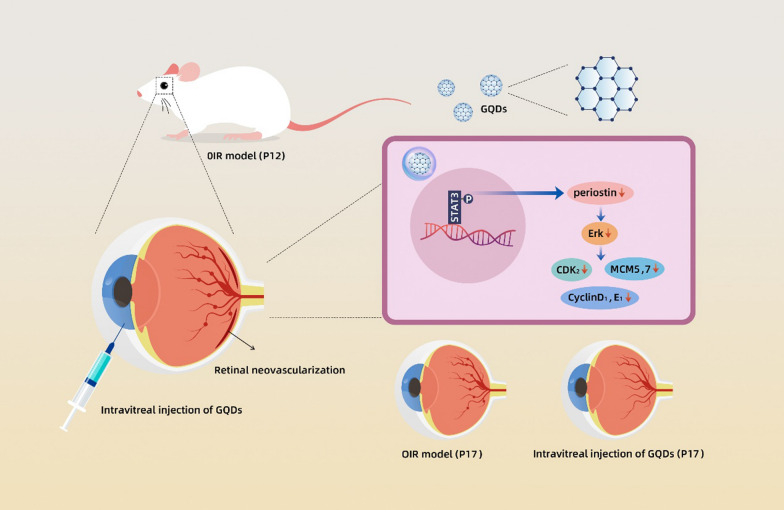

**Supplementary Information:**

The online version contains supplementary material available at 10.1186/s12951-022-01362-4.

## Background

Diabetic retinopathy, retinopathy of prematurity and central retinal vein occlusion share the common pathological process named pathological retinal neovascularization, which is the major cause of severe vision loss and even blindness nowadays [1]. Current treatments for retinal neovascular diseases include laser photocoagulation, vitrectomy and intravitreal injection of cortisol or anti-VEGF etc [2]. However, they are far from satisfaction, as decreased visual sensitivity, vitreous hemorrhage and retinal detachment often occur [3, 4]. So far, debate continues about the strategies for the management of retinal neovascularization related diseases. Hence, there remains a demand for exploring novel approaches for pathological retinal neovascularization.

Over the last few decades, nanomaterials are extensively researched, and nanotechnology has been applied extensively in biology, medicine, electronic engineering and other fields [5, 6]. It has been reported that nanomaterials have significant impact on angiogenesis due to their small size, high reactivity, and large surface area, including metal nanomaterials, silica-based nanomaterials and carbon-based nanomaterials [7]. Nowadays, carbon-based nanomaterials show great potential in nanomedicine for their unique chemical and physical properties, including carbon nanodots, carbon nanotubes, graphene, fullerenes, multi-walled carbon nanotube and carbon nanofibers [8–11]. Graphene, a new two-dimensional carbon nanomaterial, is highly biocompatible and shows excellent stability, which is considered as a novel and promising nanomedicine [12, 13].

Different from conventional graphene oxide, graphene quantum dots (GQDs) are a novel nanomaterial made from few-layer graphene sheets (below 20 nm), which show wider application compared with graphene or graphene oxide (GO) [14, 15]. GQDs are able to penetrate through biological membranes and exhibit excellent biocompatibility, fluorescence properties, and superior physiological stability, which attract extensive interest within the field of bioimaging and targeted drug delivery [16, 17]. Furthermore, recent evidence suggests that GQDs exert a direct anti-tumor efficacy toward breast cancer in vitro and in vivo [18, 19]. Also, it is reported that GQDs could penetrate the blood brain barrier (BBB) and have an inhibitory effect on fibrillization of α-syn, triggering fibril disaggregation in Parkinson’s disease [20, 21]. Pathological retinal angiogenesis shares some similarity with tumor such as uncontrolled cell growth and cell migration. Meanwhile, there is blood ocular barrier to avoid toxic substances. The previous investigations indicate that GQDs are able to penetrate BBB and suppress tumor growth, which indicates GQDs possess the potential to overcome the blood ocular barrier and to disrupt pathological retinal angiogenesis.

Periostin is a secreted matricellular protein, as well as an extracellular matrix (ECM) protein. It plays important roles in the development of bones, skins and heart, and involves in pathogenesis of various inflammatory diseases and tumor metastasis [22, 23]. Study shows that periostin is associated with the progression of hepatocellular carcinoma and it could activate hepatic stellate cells through integrin–FAK–STAT3–periostin pathway [24]. In the present study, we found that GQDs could inhibit angiogenesis in vitro and in vivo, and the transcriptomic analysis showed that RNA level of periostin and cell cycle-related proteins were different expressed in GQDs group. Western blotting demonstrated that the expression of periostin and cell cycle-related proteins was decreased after treatment by GQDs. GQDs inhibit the expression of periostin via STAT3, and further regulated cell cycle-related protein levels through Extracellular signal regulated kinase (ERK) pathway. The signaling pathway was conformed in vivo using OIR mouse model. These data suggested that GQDs could be a novel anti-angiogenic drug for the treatment of pathological retinal angiogenesis.

## Results

### Characterization of GQDs

**Fig. 1:**
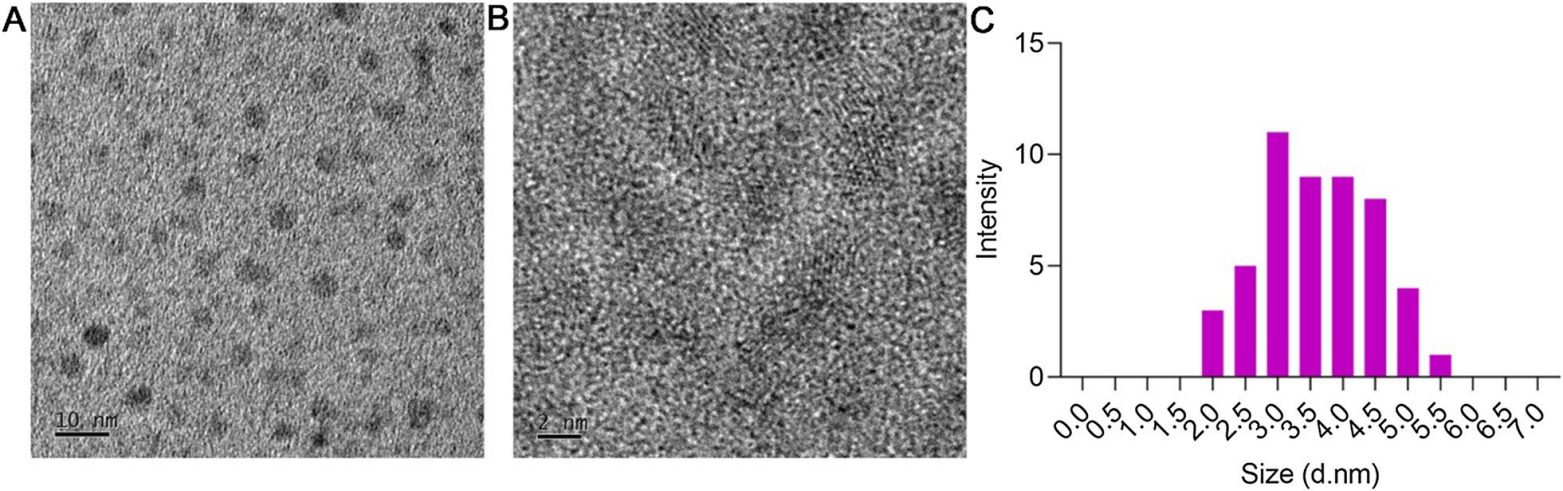
Characterization of GQDs. **A**, **B** TEM image with different magnification of GQDs. **C** size distribution of GQDs

GQDs were purchased from Nanjing XFNANO Materials Tech Co Ltd (XF152, Nanjing, China). The microstructure of GQDs were characterized using TEM microscope. The GQDs were uniformly dispersed and showed spherical morphology (Fig. 1A, B). As shown in Fig. 1C, the size distribution of GQDs ranged from 1 to 7 nm and the mean is 3.6 nm.

### The effect of GQDs on cell proliferation

The biocompatibility of GQDs is of great importance for subsequent experiments. Thus, we first assessed the viability of HUVECs during a 24 h incubation with GQDs at 0, 12.5, 25, 50, and 100 µg mL^−1^. The viability of HUVECs with GQDs at 12.5 µg mL^−1^ showed no significant difference compared with GQDs at 0 µg mL^−1^, while exposure of HUVECs to GQDs at 25, 50 and 100 µg mL^−1^ could inhibited the growth of HUVECs dose-dependently (Additional file 1: Fig. S1A). GQDs at 25 and 50 µg mL^−1^ were chosen as the appropriate concentration for follow-up experiments. To further test the biocompatibility of GQDs, we performed cell live/dead assay. HUVECs were treated with GQDs for 24 h, then stained with Calcein-AM (CAM) and Propidium Iodide (PI) (Additional file 1: Fig. S1B). The percentages of HUVECs stained by PI were 1.1%, 1.0% and 1.1% respectively, showing no statistical difference among these three groups. The data showed that 25 and 50 µg mL^−1^ GQDs did not induce apparent cell toxicity.

**Fig. 2:**
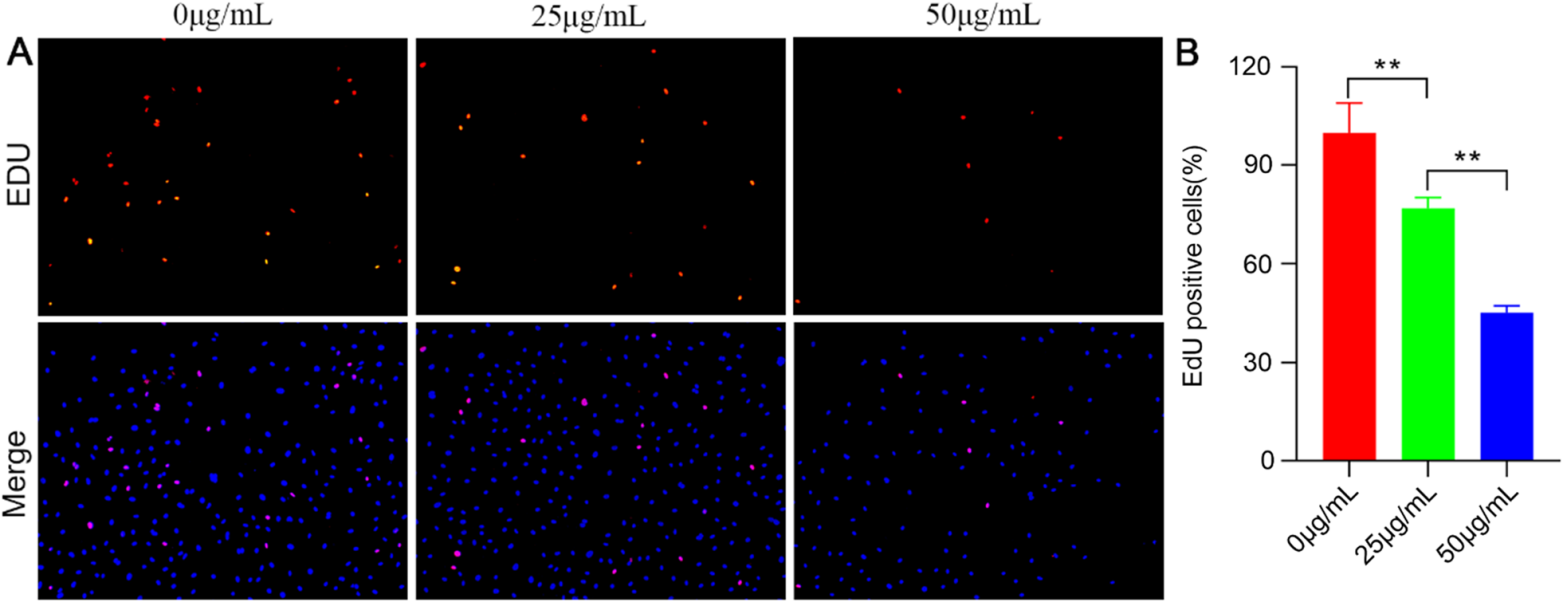
GQDs inhibit HUVECs cell proliferation in vitro. **A** GQDs inhibit the proliferation of HUVECs. EdU assay was used to determine cell proliferation. Total cells were stained in blue and proliferating cells were stained in red. **B** quantitative measurements of the percentage of EdU positive cells. Data was presented as the means ± SD, n = 4 (**P < 0.01; ***P < 0.001)

Furthermore, we conducted EdU assay to explore the effect of GQDs on cell proliferation (Fig. 2A). HUVECs were treated with GQDs at 0, 25 and 50 µg mL^−1^ for 24 h and then stained by EdU cell proliferation assay kit. The result showed that the percentage of EdU positive cells in 25 µg mL^−1^ GQDs group and 50 µg mL^−1^ GQDs group decreased dose-dependently compared with control group (Fig. 2B). The data indicated inhibitory effect of GQDs on HUVECs cell proliferation.

### GQDs impair cell migration of HUVECs

**Fig. 3:**
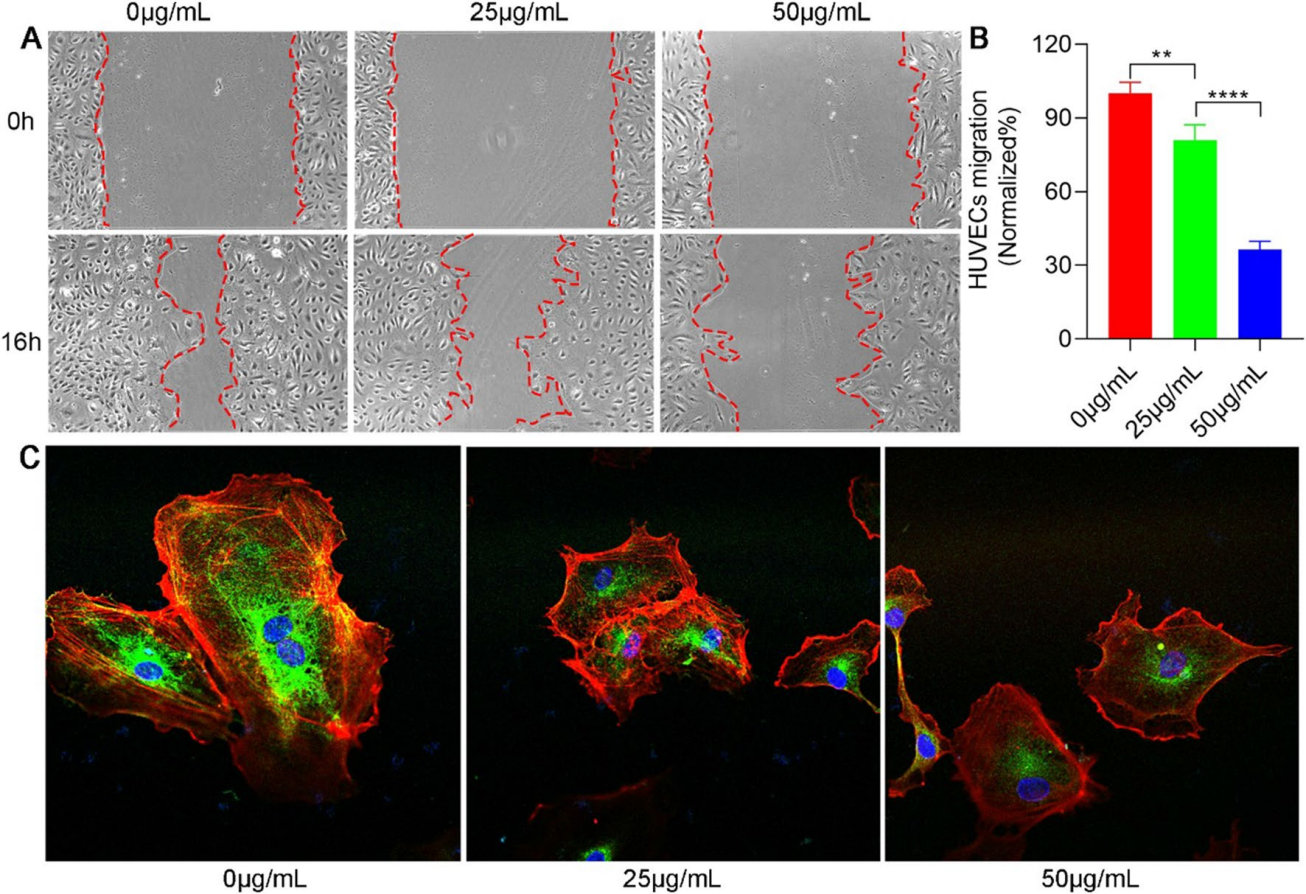
GQDs impair cell migration of HUVECs. **A** Microscopy images of HUVECs treated with GQDs in a cell scratch migration assay. **B** Quantitation of HUVECs migrated distance from the images presented in panel. Error bars represent mean ± SD. *P < 0.05, **P < 0.01. **C** Disruption of cytoskeleton in HUVECs by GQDs. Actin and tubulin were visualized using phalloidin-rhodamine (red) and anti-α-tubulin antibody (green), respectively. Data was presented as the means ± SD, n = 4 (**P<0.01; ***P < 0.001)

To explore whether GQDs affected the cell migration of HUVECs, a wound healing assay was performed. HUVECs were seeded in twenty-well plates and wounded by a sterile pipette tip. After cocultured with GQDs (0, 25 and 50 µg mL^−1^) for 16 h, the scratch region of HUVECs nearly disappeared in the control group under the microscope. In contrast, 25 µg mL^−1^ GQDs group and 50 µg mL^−1^ GQDs group still exhibited obvious wounds in a dose-dependent manner, suggesting that the migration of HUVECs was inhibited when exposed to a concentration gradient of GQDs (Fig. 3A, B).

The changes in cytoskeleton assembly are critical for cell migration. Thus, we used phalloidin and α-tubulin antibodies to stain the cytoskeleton of HUVECs to observe whether GQDs affected the cytoskeleton of HUVECs (Fig. 3C). Immunofluorescence confocal laser scanning microscopy images indicated that GQDs could impair cytoskeletal assembly in HUVECs, contributing to decreased migration of HUVECs. All together, these results demonstrated that GQDs could efficiently impair cell migration of HUVECs in vitro.

### GQDs attenuate tube formation and sprouting of HUVECs

**Fig. 4:**
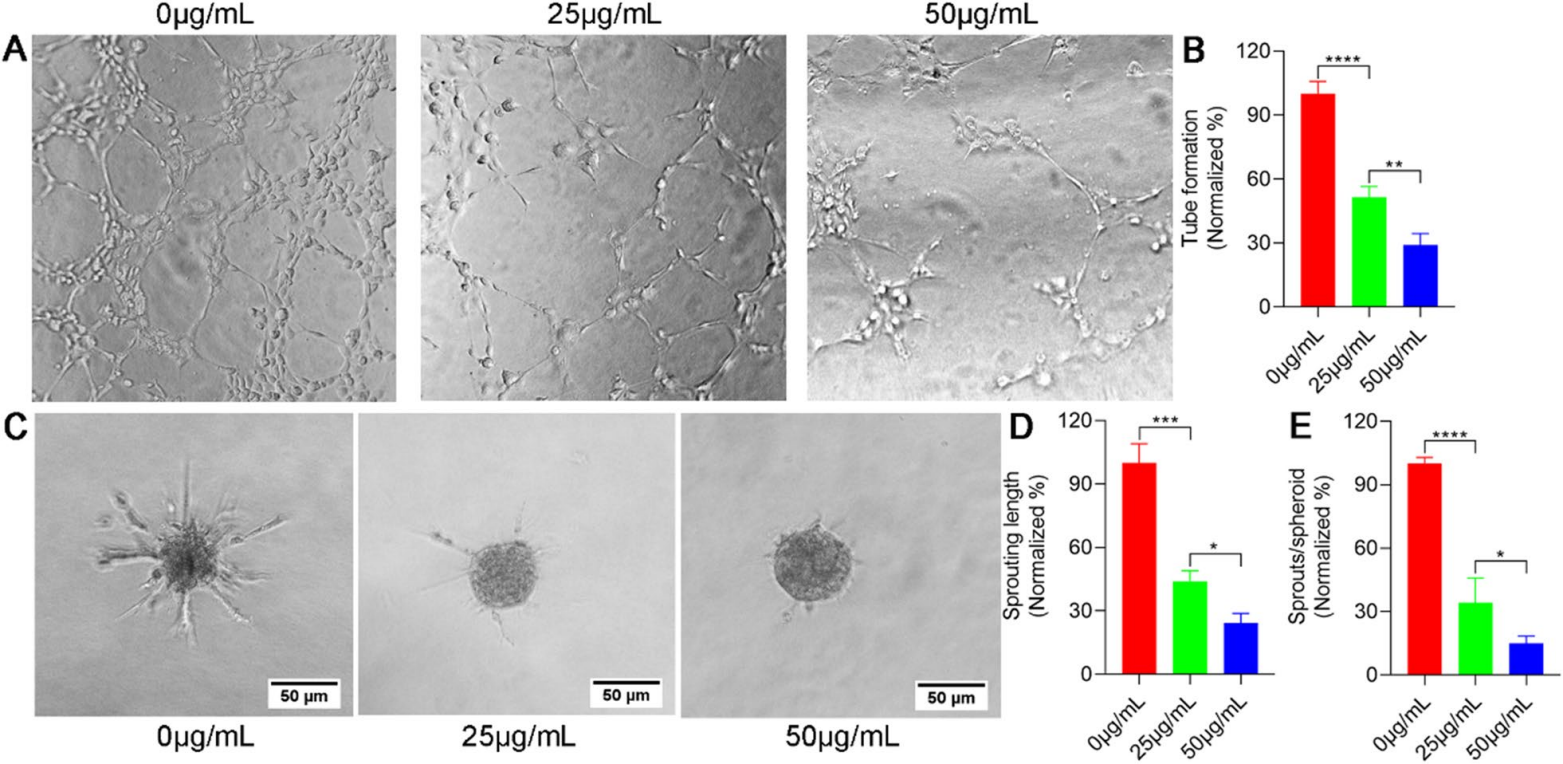
GQDs attenuate tube formation and sprouting of HUVECs. **A** Representative images of GQDs inhibited tube formation of HUVECs. **B** Quantitative of the relative tube formation rate for each group. **C** Representative images of GQDs inhibited angiogenesis in sprouting assay. **D**, **E** HUVECs sprouting was quantified as sprouting length of HUVECs and sprouts numbers surrounding the spheroid. Error bars represent mean ± SD. *P < 0.05, **P < 0.01

To evaluate the ability of GQDs to attenuate angiogenesis in vitro, we performed a tube formation assay on Matrigel. As shown in Fig. 4A, B, the number of capillary-like structures decreased apparently in GQDs treated groups than that in control group. To further verify the anti-angiogenic ability of GQDs in vitro, we analyzed HUVECs sprouting using the sprouting assay. The data showed that the ability of HUVECs to sprout was significantly attenuated by GQDs (Fig. 4C–E). Taken together, the above cell function experiment showed that GQDs could inhibit endothelial cells angiogenesis in vitro dose-dependently.

### GQDs inhibit retinal angiogenesis in the OIR model

**Fig. 5:**
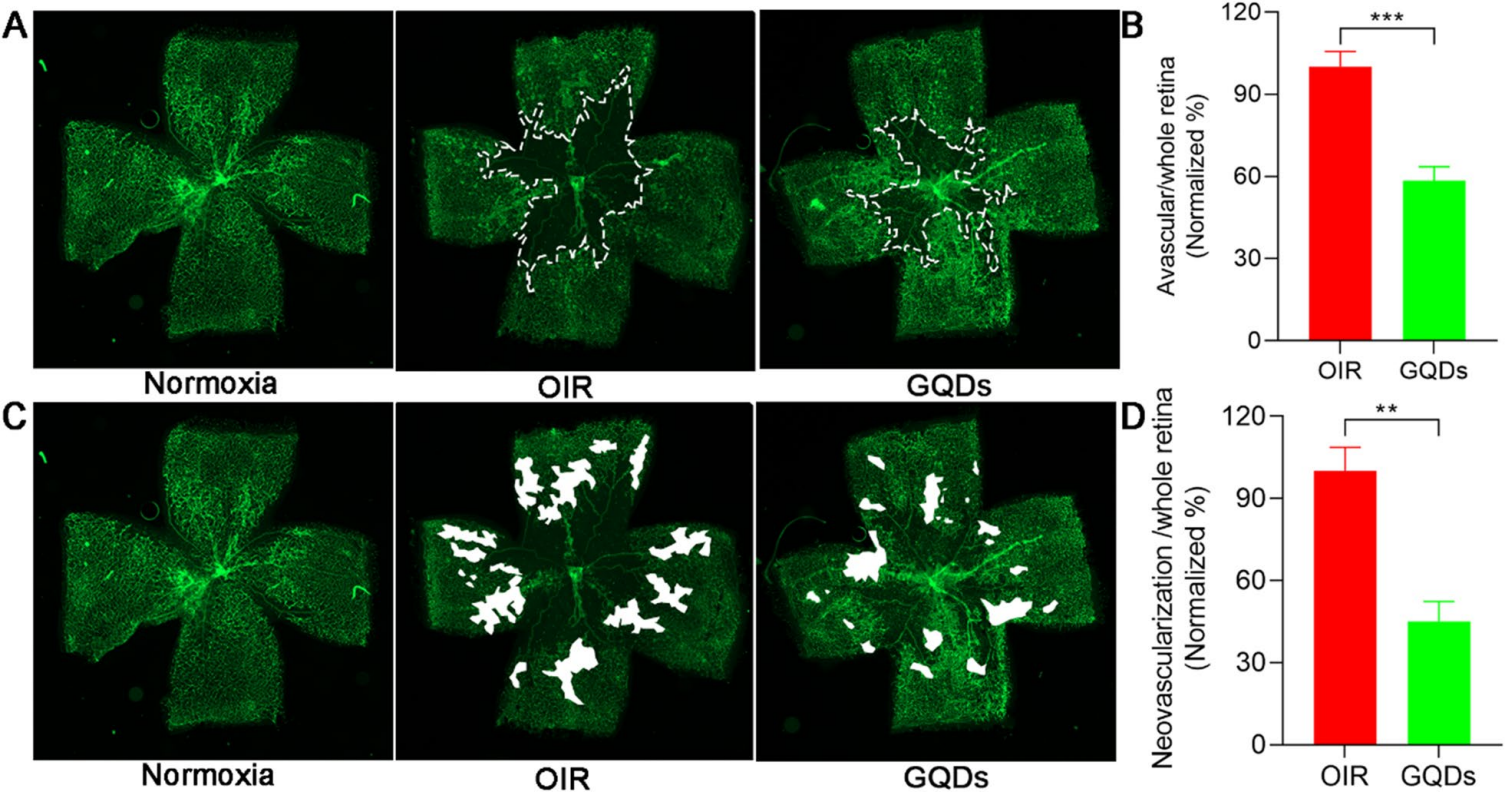
GQDs inhibit retinal angiogenesis in the OIR model. **A** Representative images of the mouse retinas from normal mouse, OIR control group and OIR models injected with GQDs. The white dotted line outlines avascular area in the central retina. **B** Quantitative analysis of the avascular area in OIR control group and GQDs treated group. **C** Representative images of the mouse retinas from normal mouse, OIR control group and OIR models injected with GQDs. The white part represents the neovascular tufts in retina. **D** Quantitative analysis of the neovascular area in OIR control group and GQDs treated group. Data was presented as the means ± SD, n = 4 (**P < 0.01; ***P < 0.001)

To further evaluate the effect of GQDs on angiogenesis in vivo, we established oxygen induced retinopathy model (OIR model), in which hyperoxia caused significant vessel loss and then room air caused hypoxic avascular retina to trigger normal blood vessels regeneration and retinal neovascularization. The retinas were collected and fixed at P17 and FITC-isolectin B4 (IB4) was used to stain retinal vessels to quantify the nonperfusion area and neovascular areas. The results showed that compared with normal retinal vasculature of mice, there were significant nonperfusion area and neovascular tufts in retina of OIR model (Fig. 5A and C). Subsequently, we performed intravitreal injection with GQDs (1 mg mL^−1^,2µL) and PBS (2µL) in OIR model at P12 respectively. The toxicity in vivo was evaluated by hematoxylin and eosin staining. The data indicated that GQDs were biocompatible in vivo (Additional file 1: Fig. S2). Then, the retinas were collected, fixed and stained as above. Notably, intravitreal injection with GQDs could significantly decrease nonperfusion area and neovascular areas of OIR mice compared with injection with PBS (Fig. 5A–D), indicating GQDs could inhibit retinal neovascularization in vivo in OIR model.

### Analysis of gene expression profiles in GQDs treated HUVECs

**Fig. 6:**
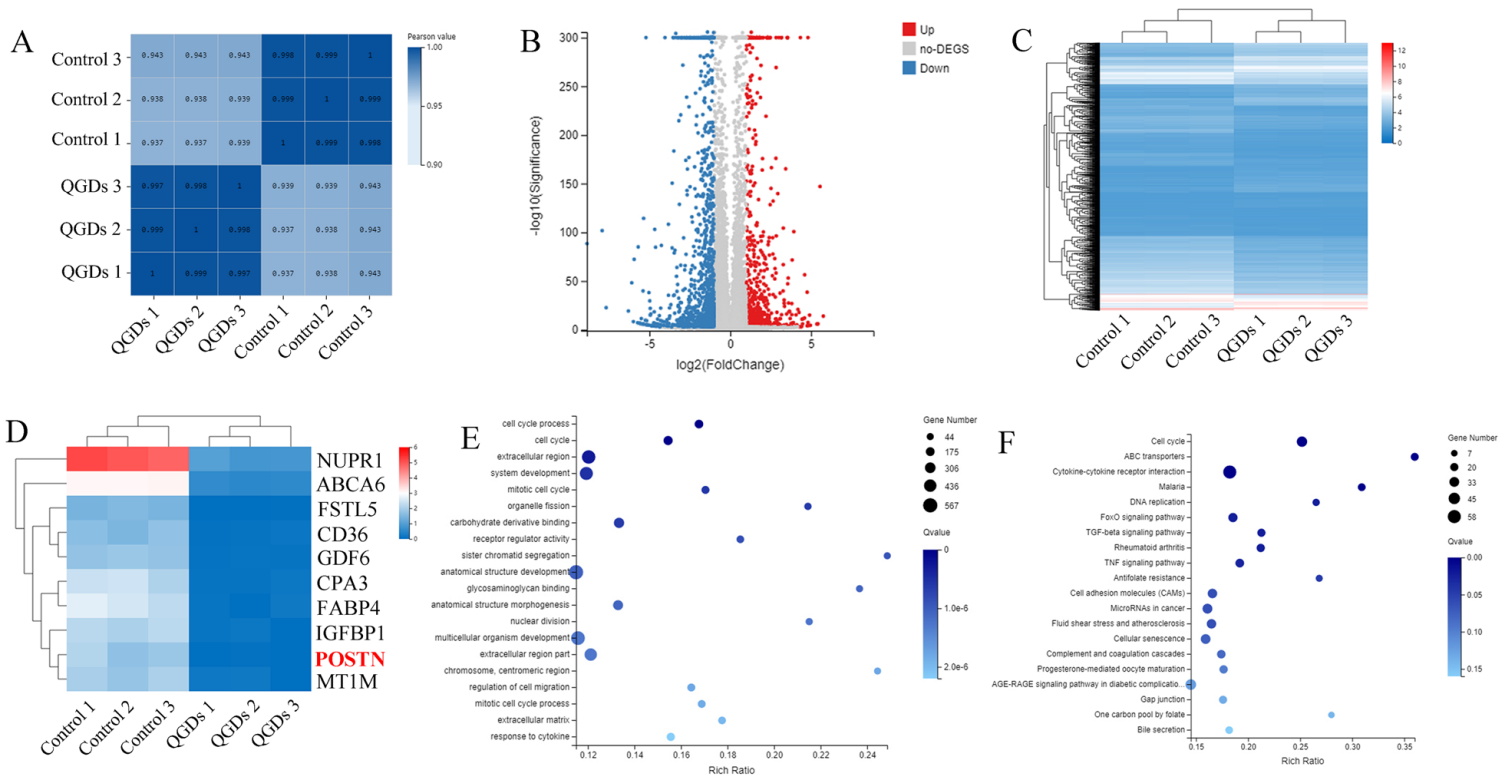
Analysis of gene expression profiles in GQDs treated HUVECs. **A** Pearson correlation coefficient of GQDs groups and control groups. **B** Log2 fold change for GQDs treatment compared to the control. **C** Heatmap of hierarchical clustering of interested genes. Blue and red indicate down-regulation and up-regulation respectively. **D** Heatmap of hierarchical clustering of the top 10 differentially expressed genes. **E** GO categorization of the top 10 differentially expressed genes. **F** KEGG pathway enrichment analysis of the top 10 differentially expressed genes

To further identify the mechanisms of GQDs inhibiting angiogenesis, we next performed RNA sequencing analysis of HUVECs in control and GQDs treated groups. Firstly, we calculated the Pearson correlation coefficient of data from three GQDs treated groups and three control groups, finding that significant differences existed in the correlations between GQDs groups and control groups (Fig. 6A). A total of 16,380 genes were identified, among which 1817 genes were screened to be differentially expressed in GQDs treated groups compared to control group (678upregulated genes and1139 downregulated genes) (Fig. 6B and C). Periostin (postn) belongs to the top ten differentially expressed genes after treatment with GQDs (Fig. 6D). Gene ontology (GO) analysis was implemented to analyze the function of the differently expressed genes. As shown in Fig. 6E, the genes were enriched for cell cycle process and cell cycle. Meanwhile, we performed pathway enrichment analysis of significant proteins through Kyoto Encyclopedia of Genes and Genomes (KEGG), finding that cell cycle was also involved in the major pathways (Fig. 6E). Therefore, we proposed the assumption that periostin and cell cycle-related proteins were associated with the inhibitory effects of GQDs on angiogenesis.

### GQDs induce cell cycle arrest in G1 phase

**Fig. 7:**
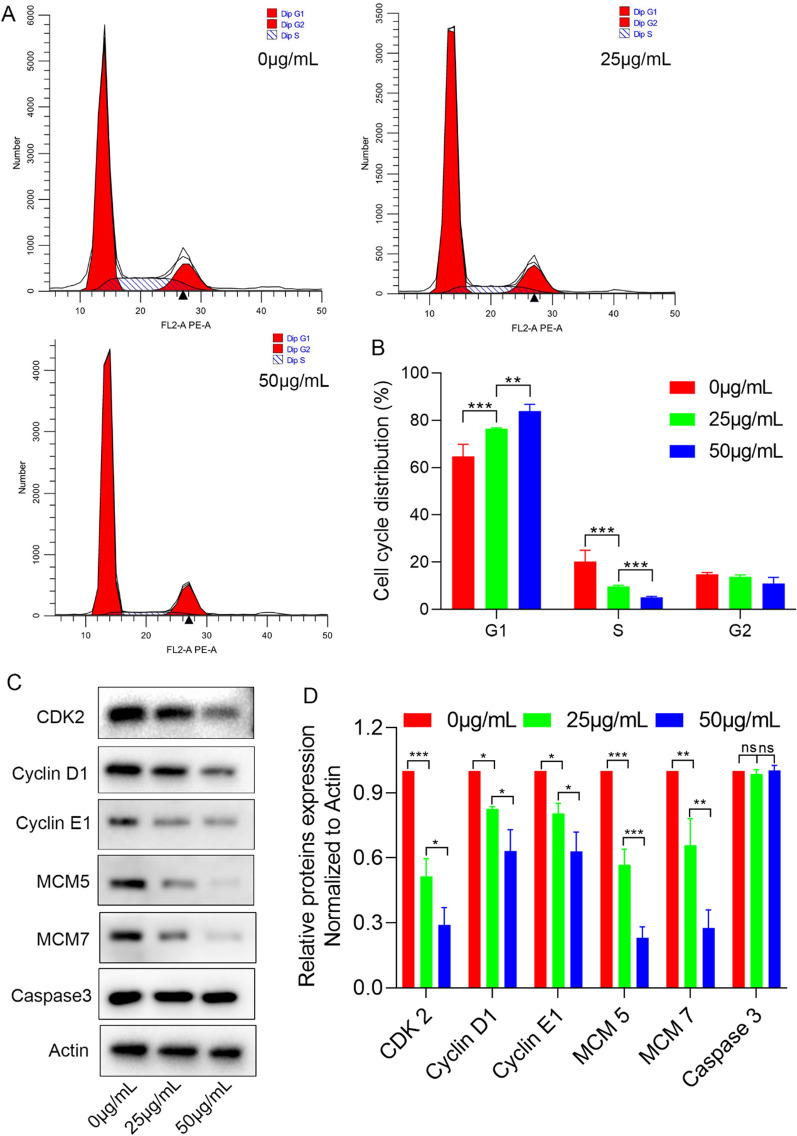
GQDs induce cell cycle arrest in G1 phase. **A** Representative images of HUVECs cell cycle distribution in the control group and GQDs group. **B** Quantitative analysis of cell cycle distribution. Data was presented as the means ± SD, n = 3 (**P < 0.01; ***P < 0.001). **C** Electrophoresis results of the cell cycle-related proteins, CDK2, Cyclin D1, Cyclin E1, MCM5, MCM7 and Caspase 3. **D** Quantitative analysis of relative proteins expression. Data was presented as the means ± SD, n = 3 (*P < 0.05, **P < 0.01; ***P < 0.001)

Generally, cell cycle can be divided into three phases, G0/G1 phase, S phase, and G2/M phase according to DNA synthesis [25]. Flow cytometry was used to analyze the cell cycle change of HUVECs after the cells were treated with GQDs for 24 h. As is showed in Fig. 7A, B, GQDs could significantly induce HUVECs cell cycle arrest at G1 phase with a decreased distribution in S phase. Sequential activation of cyclins and cyclin-dependent kinases is involved in the regulation of the cell cycle [26]. To verify whether the cell cycle-related proteins were involved in GQDs induced cell cycle arrest, we conducted western blotting. As revealed in Fig. 7C, the expression of CDK2, CYCLIN D1, CYCLIN E1 were decreased in GQDs treatment groups compared to control. Minichromosome maintenance (MCM) proteins play an important role in promoting DNA replication [27]. We also found that GQDs could inhibit the expression of MCM5 and MCM7 in HUVECs (Fig. 7C). Meanwhile, there was no differential expression of the apoptosis-associated proteins named caspase 3 (Fig. 7C, D). Collectively, the results above suggested that GQDs effectively inhibited cell cycle progression via inducing cell cycle arrest at G1 phase by a reduction in the expression cell cycle-related proteins in HUVECs, while apoptosis-related protein was not affected.

### GQDs inhibit the expression of periostin by downregulating p-STAT3

**Fig. 8:**
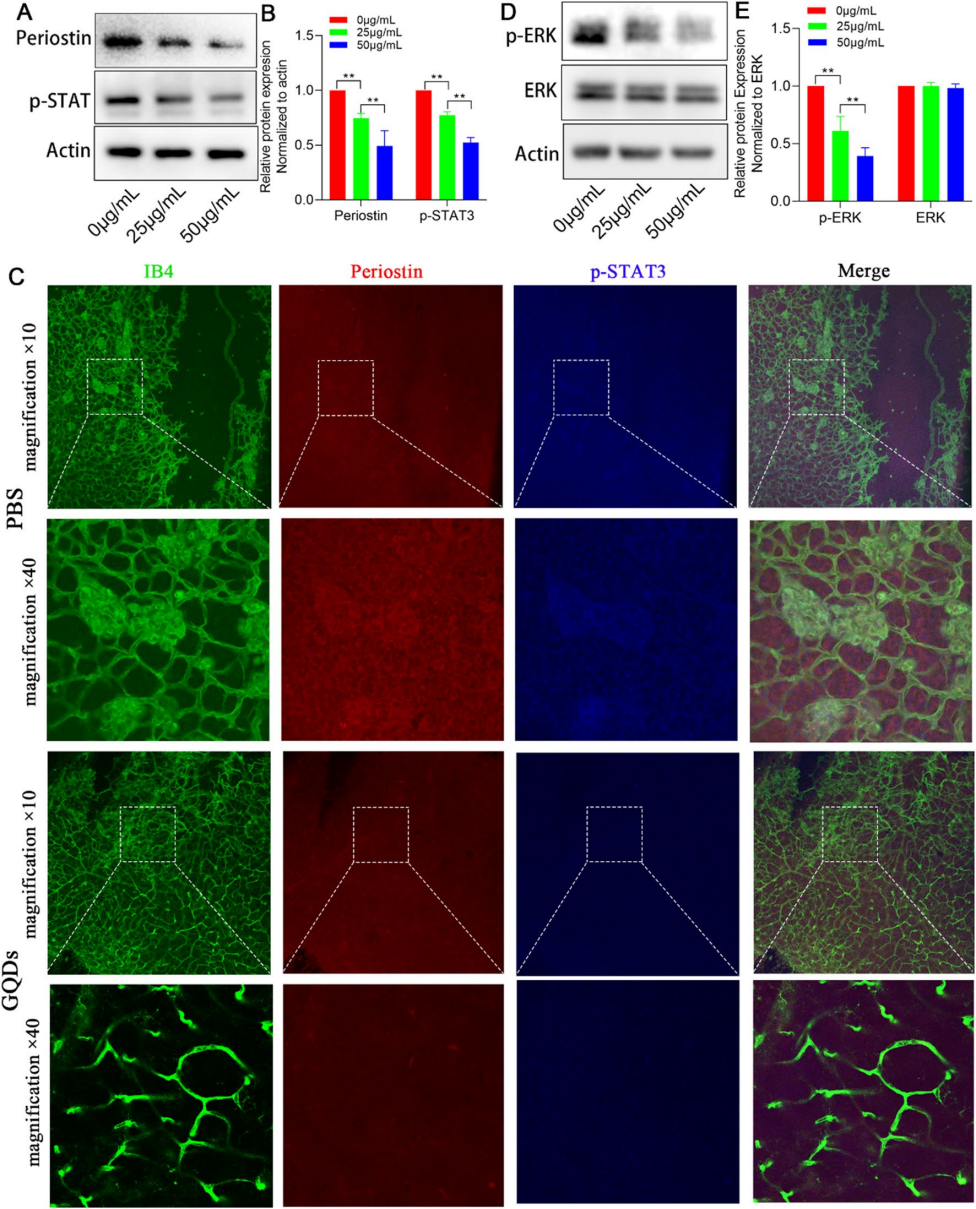
The possible mechanism of GQDs rescued angiogenic retinopathy. **A** GQDs inhibit the expression of periostin by downregulating p-STAT3. **B** Quantitative analysis of relative proteins expression. Data was presented as the means ± SD, n = 3 (**P < 0.01). **C** Representative confocal images of OIR model retinas after intravitreal injection with PBS and GQDs. Retinas were stained with IB4 (green), periostin(red) and p-STAT3 (blue). **D** GQDs inhibit the phosphorylation of ERK. **E** Quantitative analysis of relative proteins expression. Data was presented as the means ± SD, n = 3 (**P < 0.01)

RNA sequencing analysis revealed the inhibited expression of preiostin in GQDs group compared with control group. Therefore, western blotting was performed to analyze the expression of periostin in GQDs treated HUVECs and control group. Consistent with the result of gene expression profiles, the level of periostin in GQDs treated groups decreased significantly, which suggested GQDs might inhibit retinal angiogenesis through suppressing the expression of periostin (Fig. 8A–B). STAT3 is a transcription factor, which controls the expression of periostin [28]. Then, we analyzed the expression of p-STAT3 by western blotting. In line with the aforementioned studies, we found that a decreased expression of p-STAT3 following the treatment with GQDs (Fig. 8A–B). Taken together. These data confirmed that STAT3- periostin signaling pathways was disrupted in the process of GQDs inhibiting angiogenesis.

Additionally, the results of immunofluorescence assay in vivo also supported the hypothesis. We first observed the expression of periostin in retinal vascular. The data showed that the expression of periostin co-localized with IB4 stained retinal blood vessels, and periostin was highly expressed in neovascular tufts in the OIR model (Additional file 1: Fig. S3A). The data suggested that periostin was up-regulated in the blood vessels in OIR model, which could be a target for intervention of pathological angiogenesis. After intravitreal injection of GQDs, the fluorescence signals of both p-STAT3 and periostin were decreased in neovascular areas compared with PBS groups (Fig. 8C), which was consistent with the data in vitro. These data suggested that GQDs could inhibit angiogenesis by inhibiting STAT3- periostin signaling pathways in vitro and in vivo.

Previous studies indicated that there was a close association between periostin and cell cycle-related proteins. It was reported that periostin silencing down-regulated expression of cyclins E2, A2, CDK1, CDK 2, and CDK 6 though ERK pathway in lung fibroblasts [29]. Based on our gene sequencing analysis that Postn and cell cycle-related genes were differently expressed in GQDs group, we presented our hypothesis that periostin might control cell cycle via regulation of ERK pathway in GQDs group. Thus, we performed western blot analysis to quantify the expression of ERK pathway. The expression of p-ERK in HUVECs treated with GQDs was decreased (Fig. 8D, E). Altogether, the data indicated that GQDs could inhibit angiogenesis in vitro and in vivo by disrupting p-STAT3/periostin /p-ERK pathway and subsequent cell cycle.

## Discussion

At present, laser photocoagulation and anti-VEGF intravitreal injection are the major Clinical treatments for retinal neovascularization, but there are still some defects. Finding an effective angiogenesis inhibitor remains a subject of intensive research due to no completely safe and long-term effective treatment for retinal neovascularization currently. In previous study, our team have found that Gold Nanorods could suppress angiogenesis by affecting ECs cell division, and that cuprous oxide nanoparticles features anti-angiogenic agents through down regulating the expression of VEGFR2 [30, 31]. These findings showed the anti-angiogenic potentials of metal and metal oxide nanomaterials, yet the relatively larger particle size might lead to adverse effects. GQDs possess smaller size, exceptional physicochemical properties and high biocompatibility, which has received extensive attention in biomedical applications [32]. It is reported that GQDs could induce apoptosis and inflammatory response in macrophages by upregulating inflammation [33]. There are also studies revealing the potential of GQDs in photodynamic therapy for cancer treatment [15]. To our best knowledge, the anti-angiogenic role of GQDs has not been reported before. This is the first report demonstrated that GQDs could inhibit angiogenesis in vitro and in vivo through down-regulating periostin expression.

The biocompatibility of NPs was a prerequisite for the future clinical application. Recently studies have shown that many metal nanoparticles and metal oxides nanomaterials possess anti-angiogenesis activities [31, 34, 35]. Nevertheless, it is reported that metal nanoparticles and metal oxides nanomaterials have potential toxicity in cells or biology by causing oxidative stress, inflammation, genetic damage [36, 37]. GQDs, with lateral dimensions of less than 10 nm, is a novel nanomaterial with many advantages, such as good biocompatibility, high water solubility and photostability [15]. Experiments by Tanveer et al. demonstrated that GQDs have minimal toxicity to cells cultured in vitro and in vital organs of rats [38]. Given problems of biocompatibility, we performed cell live/dead assay, the results of which showed its safety in anti-angiogenic applications. Owing to their extreme small size, GQDs could penetrate the biological membranes and be cleared rapidly through the kidneys. Many researches proved that GQDs was a nanomaterial with low cytotoxicity in vitro and in vivo [16, 17]. GQDs can enter the cells very easily through caveolae-mediated endocytosis and tend to accumulate in ER and nucleus, due to their smaller lateral size. Compared with GO, GQDs induce less internal cellular reactive species (ROS) level and damage to mitochondrial membranes potential. That is the reason that GQDs are relatively easier to be untaken into cells but have lower cytotoxicity than GO sheets [39].

It is well known that the angiogenesis of ECs in vitro includes cell proliferation, migration, and ability to form capillary-like tube structure [40]. Our results of cell proliferation, cell migration assay, capillary-like tube formation and sprouting assay demonstrated that GQDs had significant anti-angiogenic potential in vitro in a dose-dependent manner. The OIR model is a classic model for studying pathological neovascularization after retinal ischemia [41]. We established OIR model to evaluate the effect of GQD on retinal neovascularization. There was a robust reduction of nonperfusion area and neovascular areas in the retina of mouse injected with GQDs compared with that of mouse injected with PBS, showing a significant inhibition of pathological retinal angiogenesis by GQDs.

Periostin, a secreted matricellular protein, plays a crucial role in cell proliferation, differentiation and migration [42]. Nowadays, studies revealed that periostin is associated with multiple cancers such as colon, pancreatic, ovarian, breast cancer [43]. Microarray analysis of gene expression of HUVECs treated with GQDs revealed decreased expression of periostin, which may illustrate the underlying molecular mechanism of anti-angiogenic capacity of GQDs. The result of western blotting validated that the expression of periostin was downregulated in GQDs treated cells, which was consistent with the previous sequencing results. Several studies have reported that the expression of periostin was regulated by STAT3 [28, 44]. Our data were consistent with these reports, revealing the molecular mechanisms that GQDs prevented angiogenesis in vitro and in vivo through suppressing STAT3/periostin pathway. Meanwhile, these results were further confirmed in an OIR model. In a classical OIR model, we observed that there was an increased expression of p-STAT3 and periostin in the peripheral neovascular tufts of retina. After an intravitreal injection of GQDs, the fluorescence intensity of p-STAT3 and periostin decreased significantly in neovascular areas. The data suggested that GQDs could suppress angiogenesis in an OIR model and downregulate of expression of p-STAT3 and periostin simultaneously. Thus, we proposed that the inhibition of retinal angiogenesis by GQDs was mediated through STAT3-periostin signaling pathway.

**Fig. 9:**
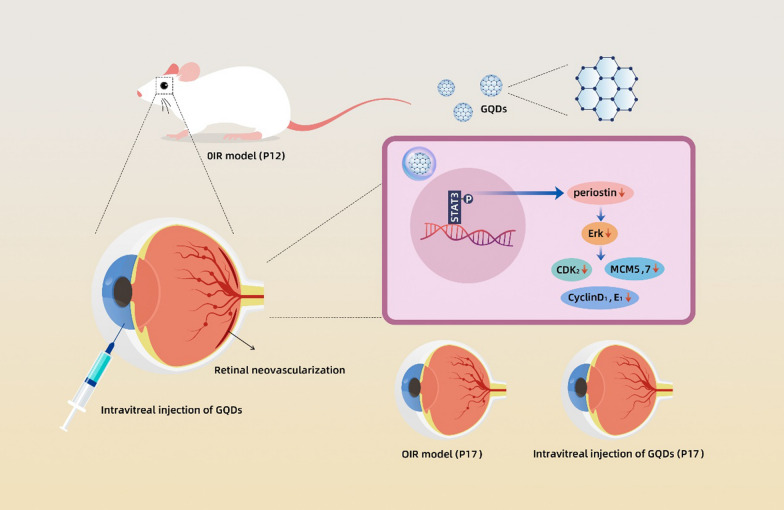
Schematic illustration of the role and mechanism of GQDs rescued angiogenic retinopathy

The cell cycle can be divided into three phases, G0/G1 phase, S phase, and G2/M phase according to DNA synthesis [25]. The EdU proliferation assay revealed that GQDs could inhibit the growth of HUVECs in vitro and RNA sequencing showed differential expression of both periostin and cell cycle-related genes. To further reveal the possible mechanism, we performed flow cytometric analysis which demonstrated that GQDs could induce cell cycle arrest at G1 phase in HUVECs. Western blotting suggested that the expression of cell cycle‐related proteins was decreased in GQDs treated groups, including CDK2, Cyclin D1, Cyclin E1, MCM5 and MCM7. Meanwhile, there was no difference in the expression of caspase 3 which was known as a key executioner of apoptosis [45]. The data further proved the biocompatibility of GQDs. Altogether, these findings indicated that GQDs could inhibit cell proliferation via regulating cell-cycle progression. As described above, studies revealed that periostin could regulate cell cycle‐related proteins expression via p-ERK signaling pathway. We analyzed the expression of p-ERK of HUVECs treated with GQDs and the results demonstrated that the expression of p-ERK was decreased in GQDs groups. Therefore, we concluded that GQDs could inhibit pathological retinal angiogenesis in vitro and in vivo by disrupting STAT/periostin/ERK pathway and subsequent cell cycle (Fig. 9).

## Conclusions

In summary, we demonstrated that GQDs could inhibit the proliferation, migration, tube formation and sprouting of HUVECs in vitro and improve pathological angiogenesis in a OIR model, which highlight the potential of GQDs as an angiogenesis inhibitor. GQDs suppressed periostin and cell cycle-related proteins expression, arresting cell cycle in the G1 phase. Furthermore, our study revealed the association between periostin and cell cycle‐related proteins after treatment with GQDS. The decreased expression of periostin could inactivate the phosphorylation of ERK to regulate cell cycle‐related proteins. These results suggested that GQDs could be a novel anti-angiogenic drug for the treatment of pathological retinal angiogenesis.

## Methods

### Materials

GQDs were purchased from Nanjing XFNANO Materials Tech Co Ltd (XF152, Nanjing, China). Antibodies to β-actin, Cyclin D1, Cyclin E1, CDK2 MCM5, MCM7, caspase 3, periostin, p-STAT were purchased from Proteintech Group (Wuhan, China). Horseradish peroxidase (HRP)- conjugated anti-mouse and anti-rabbit secondary antibodies were from Jackson ImmunoResearch (West Grove, PA, USA). Tublin, DAPI were purchased from ThermoFisher (Shanghai, China). Cell Counting Kit-8(CCK-8) was from Dojindo Laboratories, (Dojindo, Japan). Cell-light EdU Apollo567 In Vitro Kit was purchased from RiboBio (Guangzhou, China). Matrigel Matrix was purchase from BD Biosciences. (Shanghai, China). Isolectin B4 was purchased from Sigma-Aldrich (Shanghai, China). All of the cell culture plates were bought from Corning Life Sciences.

### Cell culture

HUVECs were obtained from ScienCell (San Diego, USA) and cultured in Endothelial Cell Medium (ECM, Cell Research, Shanghai, China) supplemented with 5% FBS, 1% endothelial cells growth supplement (ECGS) and 1% penicillin-streptomycin in a humidified atmosphere with 5% CO2 and a temperature of 37 °C. The cells were seeded into indicated plates for different assays when growing to 80–90% confluence.

### Cytotoxicity assay for HUVECs

The cytotoxicity of the GQDs was evaluated by the CCK-8 assay. Cells were seeded in 96-well plates at a density of 2.0 × 10^3^ cells per well and cultured in the conditions as above for 6 h. GQDs were added to the wells and cultured with the cells for 24 h. Then, the original medium was removed and 100 µL of the CCK-8 solution (10 µL CCK-8 in 90 µL ECM) was added each well and incubated for an additional 2 h. The absorbance of CCK-8 was measured by a microplate reader at a test wavelength of 450 nm with a reference wavelength of 690 nm. Cell viability (%) was equal to (OD450test−OD450blank)/(OD450control−OD450blank) × 100%. All experiments were repeated three times with three duplications.

### Cell proliferation assay

Cellular proliferation rate was measured by 5-ethynyl-2-deoxyuridine (EdU) assays as we described previously. In short, cells were seeded at a density of 2.0 × 10^3^ cells per well in 96-well plates and incubated overnight. GQDs were added into wells and incubate for 24 h. Then the culture medium was replaced with 100 uL fresh medium along with EdU (100 mM). After incubation for 2 h, the cells were stained according to the following protocol: the EdU medium was poured off and then the cells were fixed with 4% paraformaldehyde for 30 min. Then, glycine was added to wash cells for 5 min, followed by twice washes with 0.2% Trion X-100 of 100uL. Subsequently, 100 µL of Apollo fluorescent azide was added to each well to stain cells and incubated in the dark for 30 min, followed by three washes with 0.2% Trion X-100 of 100uL. Finally, Hoechst was added for another 30 min and then cells were washed with PBS three times and kept in PBS until imaging. The images were acquired and analyzed using a digital microscope system (IX81, Olympus).

### Cell migration assay

5 × 10^5^ HUVECs were added to 12-well plate and cultured with medium overnight. Scratch wounds were created across the surface of the plates using a sterile 200-µl pipette tip and the medium containing suspension cells were removed and cells were rinsed with PBS for 3 times. GQDs were added into the wells and cultured at 37 degrees with 5% CO2. 16 h later, cells were fixed with paraformaldehyde (4% in PBS) for 10 min at room temperature and subsequently washed twice with PBS. Finally, samples were observed through microscope (IX81, Olympus).

### Tube formation assay

These assays were conducted following the instructions provided by the manufacturer. Firstly, Matrigel was melted at 4 °C overnight and diluted with serum-free medium. 90 µL diluted Matrigel was added to each well of 96-well plate and solidified at 37 °C for 40 min. Then, HUVECs treated with 25 and 50 µg mL^−1^ GQDs for 24 h were digested by trypsin and then 2 × 10^4^cells were gently added to each Matrigel-coated well and incubated at 37 °C for 3 h. Images were captured using an Olympus IX81 microscope.

### Cell cycle assay

HUVECs were seeded in a 6-well plate and incubated overnight and then starved for 16 h. Subsequently, starved cells were treated with 25 and 50 µg/mL GQDs for another 72 h and then cells were trypsinized, washed twice in PBS and fixed overnight in 70% ethanol at 4 °C. The next day ethanol was removed by centrifugation and cells were washed twice with cold PBS. Finally, cells were resuspended in PBS containing ribonuclease (200 µg/ml) and propidium iodide (20 µg/ml) and incubated for 30 min. The samples were immediately analyzed by Cell Lab Quanta SC flow cytometer (BECKMAN COULTER) and data were analyzed using ModFit software.

### Western blot analysis

Total proteins of HUVECs in 6-well plates were extracted using RIPA lysis buffer (Beyotime, Shanghai, China) supplemented with protease inhibitors (20 uL/mL) and phosphatase inhibitors (20 uL/mL). Then proteins (20ul samples per lane) were separated on 10% polyacrylamide gels by SDS-PAGE and transferred onto PVDF membranes. After blocked with 5% skimmed milk for 1 h.

At room temperature, the blots were incubated with various concentrations of primary antibodies at 4 °C overnight according to the manufacturer’s protocols. Then the membranes were washed three times in TBS-T for 5 min each time and subsequently incubated with horseradish peroxidase–conjugated goat anti-mouse or anti-rabbit antibodies (1:10,000 dilution) for 2 h, followed by three washes with TBS-T 5 to 7 min each time. Lastly, the blots incubated with ECL Super Signal West Pico Chemiluminescent Substrate (Thermo Scientific) and detected using the GeneGnome HR Image Capture System (Syngene).

### Gene expression microarrays

Cells in culture dishes were treated with 50 µg mL^−1^ GQDs for 24 h and then lysed using Trizol reagent (Invitrogen) to collect total RNA. The samples were submitted to Beijing Genomics institution (Beijing, China) for mRNA sequencing and analysis. Differentially expressed mRNAs were identified by fold-change filtering (fold change > 2). Heatmaps were generated by Hierarchical Clustering with average linkage in Cluster 3.0 and visualized using Java TreeView program. Subsequently, the standard enrichment computation method was used to perform GO and KEGG pathway analyses.

### Retina preparation and analyses

The oxygen induced retinopathy (OIR) model was induced to analyze retinal angiogenesis as previously described [30]. From P7 to P12, pups and their nursing mothers were exposed to 75% oxygen and on P12 the pups were removed from oxygen supply chamber and randomly divided into two groups: GQDs-treated group and control group. 12-day-old pups were intravitreally injected with GQDs (1 mg/mL,2µL) in right eye using a 5-µL Hamilton syringe equipped with 50-gage glass capillary, and PBS (2 µL) in the contralateral eye. Five days later, the mice were euthanized by inhalation of CO_2_ and their eyes were enucleated and then fixed in 4% paraformaldehyde overnight at 4 °C. Hereafter, retinas were isolated in their entirety under a microscope and then block with 1% BSA and 0.3% Triton X-100 for 30 min at room temperature. After three times wash in PBS, the retinas were stained with FITC- conjugated Isolectin B4 and indicated primary antibody overnight at 4 °C. Then retinas were washed three times with PBS and incubated with FITC-labelled secondary antibody for 2 h at room temperature. After that, the samples were washed with PBS two times and counter-stained with DAPI (1:2000) in PBS for 30 min, followed by three PBS washes. Finally, retinas were carefully flat mounted onto glass slides and sealed with resinene. Slides were photographed using Leica TCS SP5-II confocal microscope system and the avascular areas and neovascular areas were quantified using Image J software.

### Statistical analysis

Each experiment was carried out in triplicates, and repeated at least three times. The data were first tested for normality using SPSS software (SPSS 18.0 software) and then analyzed by one-way ANOVA using Prism software (GraphPad 9.0). All results were presented as a mean ± standard deviation (SD) and the statistical differences between two groups were analyzed by unpaired Student’s t-test with statistical significance assumed at P < 0.05.

## Supplementary Information

**Additional file 1: Figure S1.** The effect of GQDs on cell viability. (A) CCK-8 assay was used to evaluate cell viability after treatment with GQDs. (B)live/dead assay was used to evaluate the toxicity of GQDs in vitro. results. Viable cells were stained in green and dead cells were stained in red with Calcein- AM/PI double dyeing kit. (C) The statistical results of live/dead assay. All data was acquired by means of ± SE from at least three independent experiments, n = 3 (**P < 0.01, ****P < 0.0001). **Figure S2.** The effect of GQDs on the morphology of retina. These was no significant changes in the morphology of retina in both groups. **Figure S3.** Representative confocal images of normal mouse retinas and OIR model retinas. Periostin was highly expression in the pathological blood vessel tissue. Retinas were stained with DAPI (blue), IB4 (green) and periostin(red).

## Data Availability

All data used to support the findings of this study are included within the article.

## Abbreviations

OIR: Oxygen induced retinopathy
GQDs: Graphene quantum dots
HUVECs: Human umbilical vein endothelial cells
BBB: Blood brain barrier
TEM: Transmission electron microscope
CAM: Calcein-AM
PI: Propidium Iodide
IB4: Isolectin B4
GO: Gene ontology
KEGG: Kyoto Encyclopedia of Genes and Genomes
EdU: 5-ethynyl-2-deoxyuridine
