# Supplementary material for: Graphene quantum dots rescue angiogenic retinopathy via blocking STAT3/Periostin/ERK signaling
Source: J Nanobiotechnology. 2022 Apr 2;20:174. doi: 10.1186/s12951-022-01362-4 (PMC8977040; doi:10.1186/s12951-022-01362-4)
Supplement: Supplementary file 1 — Additional file 1: Figure S1. The effect of GQDs on cell viability. (A) CCK-8 assay was used to evaluate cell viability after treatment with GQDs. (B)live/dead assay was used to evaluate the toxicity of GQDs in vitro. results. Viable cells were stained in green and dead cells were stained in red with Calcein- AM/PI double dyeing kit. (C) The statistical results of live/dead assay. All data was acquired by means of ± SE from at least three independent experiments, n = 3 (**P < 0.01, ****P < 0.0001). Figure S2. The effect of GQDs on the morphology of retina. These was no significant changes in the morphology of retina in both groups. Figure S3. Representative confocal images of normal mouse retinas and OIR model retinas. Periostin was highly expression in the pathological blood vessel tissue. Retinas were stained with DAPI (blue), IB4 (green) and periostin(red). [file 12951_2022_1362_MOESM1_ESM.docx]

**Graphene quantum dots rescue angiogenic retinopathy via blocking STAT3/Periostin/ERK signaling**

Na Zhao^1†,^ Xiao Gui^1†^, Qian Fang^2†^, Rui Zhang^1^, Weiye Zhu^1^, Haorui Zhang^1^, Qing Li^1^, Yukun Zhou^1^, Jiawei Zhao^1^, Xiao Cui^1^, Guangping Gao^1^, Huipeng Tang^1^, Ni Shen^1^, Taoyong Chen^2*^, Hongyuan Song^1,3*^and Wei Shen^1*^

^1^Department of Ophthalmology, Shanghai Changhai Hospital, Naval Medical University, Shanghai 200433, China.

^2^National Key Laboratory of Medical Immunology and Institute of Immunology, Naval Medical University, Shanghai 200433, China.

^3^Department of Ophthalmology, Shanghai General Hospital, Shanghai Jiao Tong University School of Medicine, Shanghai, 200080, China.

Correspondence should be addressed to Taoyong Chen: chenty@immunol.org, Hongyuan Song: hongyuansong@hotmail.com and Wei Shen: shenwzz@163.com

**
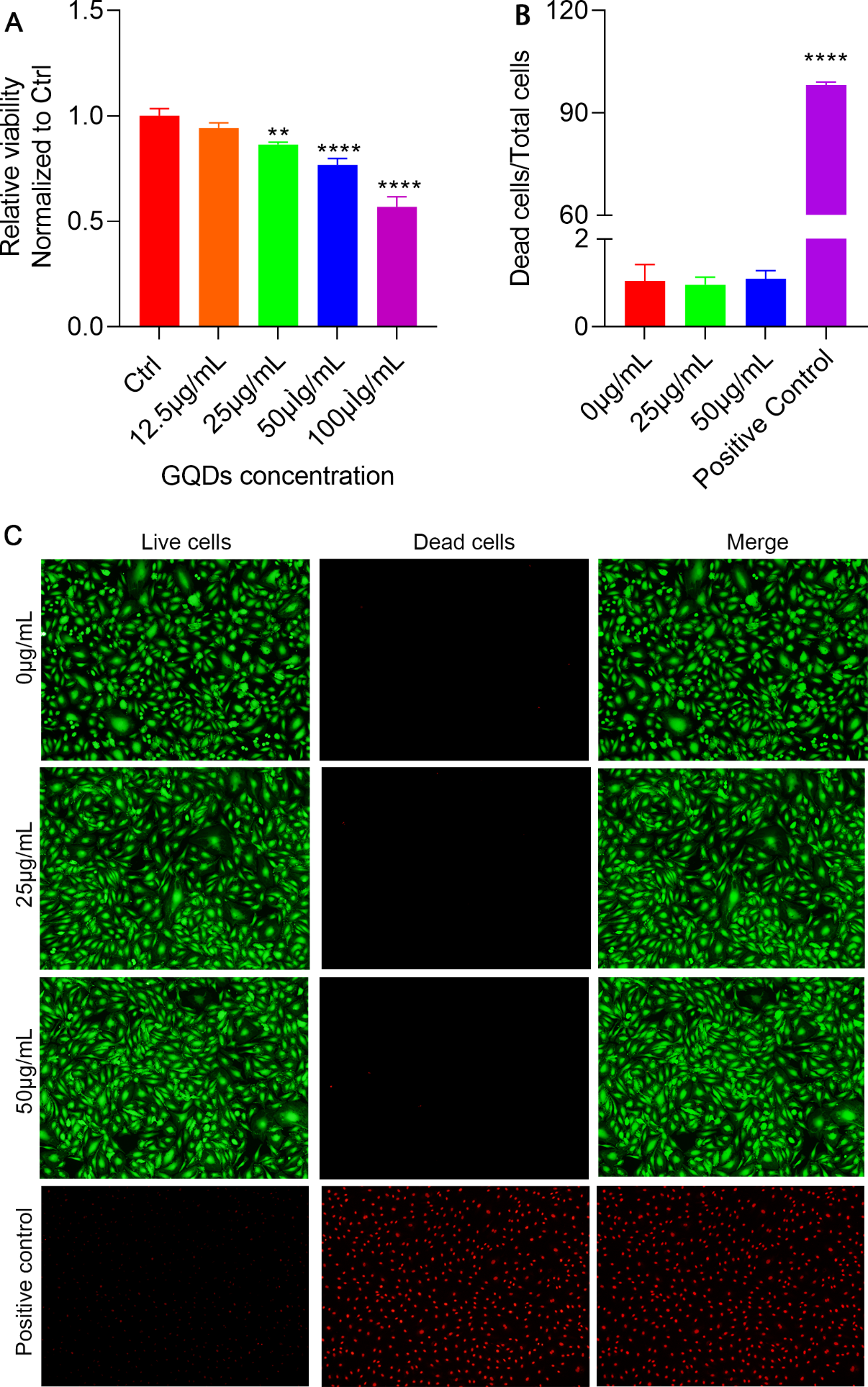
**

**Figure S1**. The effect of GQDs on cell viability. (A) CCK-8 assay was used to evaluate cell viability after treatment with GQDs. (B)live/dead assay was used to evaluate the toxicity of GQDs *in vitro*. results. Viable cells were stained in green and dead cells were stained in red with Calcein- AM/PI double dyeing kit. (C) The statistical results of live/dead assay. All data was acquired by means of ± SE from at least three independent experiments, n=3 (**P<0.01, ****P<0.0001).

**
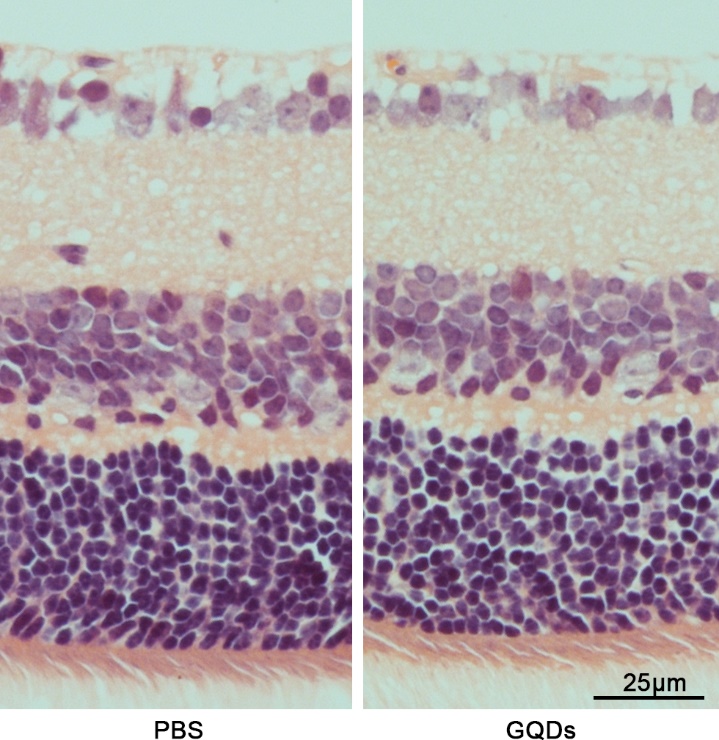
**

**Figure S2**. The effect of GQDs on the morphology of retina. These was no significant changes in the morphology of retina in both groups.

**
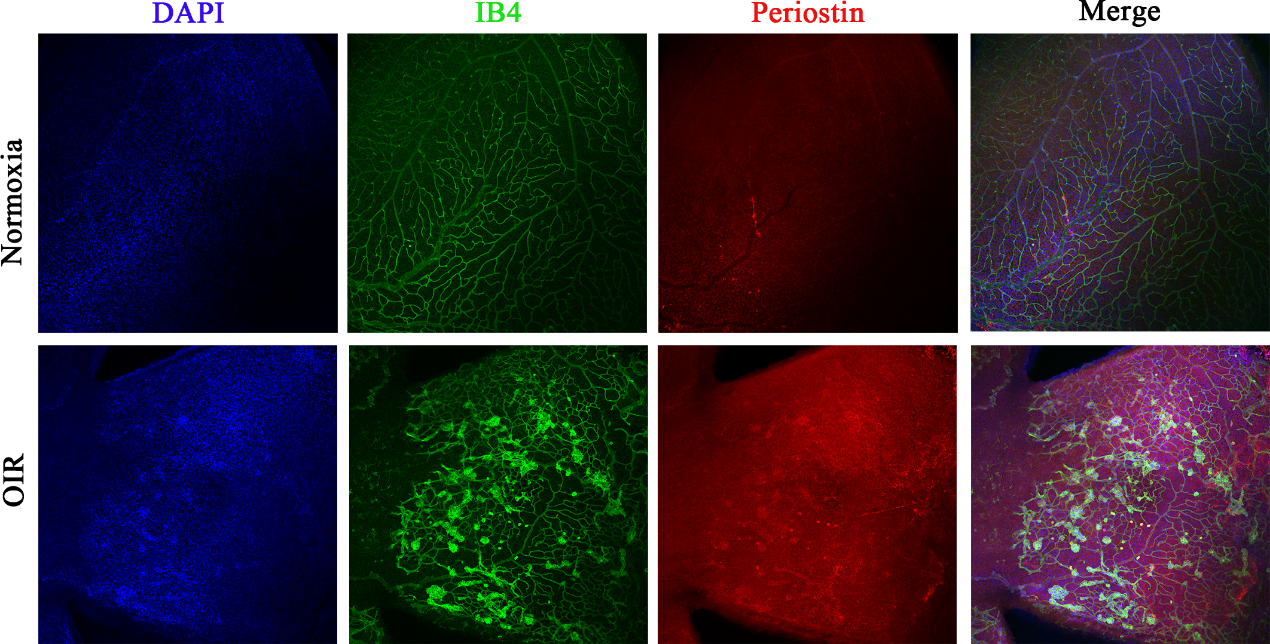
**

**Figure S3**. Representative confocal images of normal mouse retinas and OIR model retinas. Periostin was highly expression in the pathological blood vessel tissue. Retinas were stained with DAPI (blue), IB4 (green) and periostin(red).
